# Role of the long non-coding RNA PVT1 in the dysregulation of the ceRNA-ceRNA network in human breast cancer

**DOI:** 10.1371/journal.pone.0171661

**Published:** 2017-02-10

**Authors:** Federica Conte, Giulia Fiscon, Matteo Chiara, Teresa Colombo, Lorenzo Farina, Paola Paci

**Affiliations:** 1 Institute for Systems Analysis and Computer Science “Antonio Ruberti”, National Research Council, Rome, Italy; 2 Department of Biosciences, University of Milan, Milan, Italy; 3 Department of Computer, Control and Management Engineering, “Sapienza” University, Rome, Italy; Dokuz Eylul Universitesi, TURKEY

## Abstract

Recent findings have identified competing endogenous RNAs (ceRNAs) as the drivers in many disease conditions, including cancers. The ceRNAs indirectly regulate each other by reducing the amount of microRNAs (miRNAs) available to target messenger RNAs (mRNAs). The ceRNA interactions mediated by miRNAs are modulated by a titration mechanism, *i.e.* large changes in the ceRNA expression levels either overcome, or relieve, the miRNA repression on competing RNAs; similarly, a very large miRNA overexpression may abolish competition. The ceRNAs are also called miRNA “decoys” or miRNA “sponges” and encompass different RNAs competing with each other to attract miRNAs for interactions: mRNA, long non-coding RNAs (lncRNAs), pseudogenes, or circular RNAs. Recently, we developed a computational method for identifying ceRNA-ceRNA interactions in breast invasive carcinoma. We were interested in unveiling which lncRNAs could exert the ceRNA activity. We found a drastic rewiring in the cross-talks between ceRNAs from the physiological to the pathological condition. The main actor of this dysregulated lncRNA-associated ceRNA network was the lncRNA PVT1, which revealed a net biding preference towards the miR-200 family members in normal breast tissues. Despite its up-regulation in breast cancer tissues, mimicked by the miR-200 family members, PVT1 stops working as ceRNA in the cancerous state. The specific conditions required for a ceRNA landscape to occur are still far from being determined. Here, we emphasized the importance of the relative concentration of the ceRNAs, and their related miRNAs. In particular, we focused on the withdrawal in breast cancer tissues of the PVT1 ceRNA activity and performed a gene expression and sequence analysis of its multiple isoforms. We found that the PVT1 isoform harbouring the binding site for a representative miRNA of the miR-200 family shows a drastic decrease in its relative concentration with respect to the miRNA abundance in breast cancer tissues, providing a plausibility argument to the breakdown of the sponge program orchestrated by the oncogene PVT1.

## Introduction

The last years have been marked by an increasing widespread interest in non-coding RNAs, emerging as key regulators of many cellular processes in both physiological and pathological states [1–4]. This class of RNA species appears really heterogeneous, including the intensively studied microRNAs (miRNAs)—small non-coding RNAs of 20-22 nucleotides long [5–7]—as well as the most recently acknowledged long non-coding RNAs (lncRNAs). LncRNAs are non-protein coding transcripts greater than 200 nucleotides in length and lacking of extended open reading frames [8–10]. As broadly suggested by several works [11–31], lncRNAs critically participate in transcriptional and post-transcriptional regulation, though the biological functions of the majority of them largely remain to be defined yet. Recent studies have shown that some lncRNAs may have a role linked to their secondary structure [32–40], whose specific substructures can function as guide or scaffold by binding chromatin-modifying protein complexes [34, 37, 41, 42]. Although lncRNAs have low sequence conservation [43, 44], increasing evidence indicates that also their primary structure (*i.e.* nucleotides sequence) could be instrumental for their implication in a wide variety of processes, including competition for miRNA binding [30, 45–61].

Competing endogenous RNAs (ceRNAs), also known as miRNA “decoy” or miRNA “sponges”, are RNA transcripts that compete for the binding to the same miRNA *via* the base-pairing with miRNA recognition/response elements (MREs) [62–70], subsequently enabling the reduction of the amount of miRNAs available to target messenger RNAs (mRNAs). Such a mechanism of regulation of miRNA activity was firstly discovered in plants and called “target mimicry” process [71].

The first experimental evidence of lncRNAs acting as miRNA decoys modulating the derepression of miRNA targets has been found in wide variety of human cancers and specifically concerns the functioning of pseudogenes (*i.e.* copies of real genes that originate from duplications or retro-transpositions) as competitors of their ancestral genes for miRNA binding [72]. They are not translated into functional proteins because their coding potential is corrupted by premature stop codons, deletions/insertions and frameshift mutations. Nevertheless, nucleotide sequences contained within pseudogenes are well preserved, suggesting that selective pressure to maintain these genetic elements exists, and that they may indeed have an important cellular role [72]. Moreover, pseudogenes are almost as numerous as coding genes and represent a significant proportion of the transcriptome [73]. They are perfect endogenous competitors of their ancestral genes, since they retain many of the miRNA binding sites.

LncRNAs functioning as ceRNAs can be also observed in: mouse and human myoblasts, where the large intergenic non-coding RNA (lincRNA) called linc-MD1 controls muscle differentiation by targeting miR-133 and miR-135 to regulate the expression of MAML1 and MEF2C [74]; human embryonic stem cells, where linc-RoR competes with the transcription factors NANOG, OCT4, SOX2 for binding to miR-145 regulating cell pluripotency and self-renewing [75]; human thyroid cancer, where the thyroid-specific lncRNA PTCSC3 targets miR-574-5p [76]; human embryonic kidney 293 (HEK293) cells, where the lncRNA H19 modulates the let-7 miRNAs family availability causing precocious muscle differentiation [77].

Most recently, also the new-appreciate circular RNAs (circRNAs) appear to exert ceRNA activity [70, 78–80]. They are a class of non-coding RNAs derived mostly from a non-canonical form of alternative splicing, whereby the exon ends are joined to form a continuous loop [81–84]. In particular, the exonic circRNA CDR1 relieves the activity of miR-7 on its target impairing midbrain development in mammals [85] and the testis-specific cirRNA Sry serves as a miR-138 sponge [80].

In our previous work [86], we developed a purely data-driven approach focused on the identification of lncRNAs acting as new putative ceRNAs in a large set of tumour and matched-normal samples (*i.e.* tissues that are adjacent to the tumour and taken from the same patient) of breast invasive carcinoma available from The Cancer Genome Atlas (TCGA) [87, 88]. By applying a multivariate statistical analysis refined by the requirement of a seed match enrichment, we built a network of miRNA-mediated sponge interactions (MMI-networks) in both physiological and pathological states and compared the two obtained MMI-networks. We found a marked rewiring in the ceRNA program between normal and pathological breast tissues. At the heart of this phenomenon is the lncRNA PVT1 that serves as miRNA sponge in normal tissues, but not in cancer. Moreover, it revealed, in normal MMI-network, a net binding preference towards the miR-200 family, which it antagonizes to regulate the expression of hundreds of mRNAs known to be related to the cancer development and progression (*e.g.* GATA3, CDH1, TP53, TP63, TP73, RUNX1, and RUNX3).

PVT1 is a large intergenic non-coding RNA that appears to be strongly conserved between mouse and human [89–94]. The PVT1 gene [95] spans across a genome interval of over 300 kb (*i.e.* bases 128806779–129113499 within the February 2009 human genome build GRCh37/hg19) on the forward strand of chromosome 8 [96]. Moreover, PVT1 lies in a recognized cancer risk locus that it shares with MYC and shows highly complex gene architecture. Indeed, its locus gives rise to over 20 different variants of the lncRNA according to the Ensembl annotations of the human genome (release 75) and also produces a cluster of six annotated microRNAs (*i.e.* miR-1204, miR-1205, miR-1206, miR-1207-5p, miR-1207-3p, and miR-1208) [96–101]. The last years have been the scene of increasing advancements in studying PVT1 role in tumour cells [28, 96, 102–107] and its overexpression appears as a frequent event in a wide variety of cancers [98, 103, 104, 108, 109]. In addition to a putative ceRNA activity, interesting ways of functioning of PVT1 have been suggested, such as the regulation of the protein stability of the well-known MYC oncogene through its secondary structure [99, 110–112].

The miR-200 family consists of five members: miR-200a, miR-200b, miR-200c, miR-141 and miR-429. On the basis of the similarities of their seed sequences (*i.e.* 6 nucleotides at positions 2-7 from the miRNA 5’-end [113]), the miR-200 family members can be clustered into two groups only differing for one nucleotide in the seed sequence: miR-200a/141 (AACACU) and miR-200b/200c/429 (AAUACU) [114, 115]. The miR-200 family is one of the most widely studied for its crucial role in cancer initiation, metastasis, diagnosis, and treatment. A large number of studies showed that the down-regulation of the miR-200 family members appears to promote the epithelial-mesenchymal transition, proving their suppressive effects on cancer cell proliferation, migration, and invasion [115–118]. However, Park *et al*. [119] experimentally demonstrated how the down-regulation of all members of the miR-200 family would result in mesenchymal cell lines, while a their up-regulation would appear characteristic of an epithelial phenotype.

In the dataset we analysed in [86], all members of the miR-200 family appear to be highly up-regulated in cancer tissues (from 4- to 8- folds) and this up-regulation is counteracted by a similar, even if not comparable, overexpression of PVT1 that in cancer tissues appears to increase of about two folds. This observation could in principle warrant the annihilation of the PVT1 sponge activity noted in cancer dataset. In fact, Salmena *et al*. [63] suggested that the breakdown of the ceRNA activity could be due to a titration mechanism, *i.e.* large changes in the ceRNA expression levels that either overcome, or relieve, the miRNA repression on competing ceRNAs; similarly, large changes in the miRNA expression allow miRNAs to escape the recruitment accomplished by ceRNAs.

Here, we are interested to analyse the specific conditions required for a ceRNA landscape to occur, betting on the titration mechanism as the main culprit. In particular, inspiring by our amazing results of [86] and by the growing interest of the scientific community in the oncogenic role of the lncRNA PVT1, we focused on its activity as sponge modulator of the activity of the miR-200 family members on their targets and on the withdrawal of its decoy service in breast cancer tissues.

## Materials and methods

### Algorithm for identifying ceRNA-ceRNA interactions

**Fig 1:**
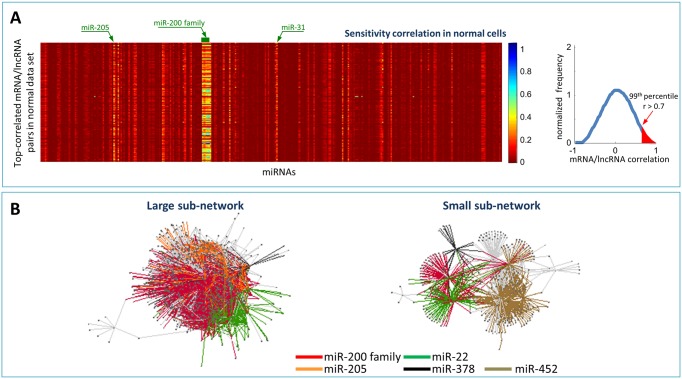
Sensitivity correlation and normal MMI-network. (A) Left: heat-map representing the sensitivity correlation, given in the Eq 1, for the top-correlated pairs (N = 87398) of mRNAs and lncRNAs (rows), previously identified in [86], in normal breast samples versus each miRNA (columns) that was expressed in the same tissues. Light vertical stripes point to a little pool of miRNAs that are responsible for the high correlation between all the top-correlated mRNA/lncRNA pairs. Colour key: red to blue scale corresponds to low to high sensitivity correlation. Right: the distribution of the Pearson correlations between mRNA and lncRNAs expression profiles. The pairs that are highlighted in red correspond to the top-correlated mRNA/lncRNA pairs: Pearson correlation values exceeding the 99^th^ percentile of the overall correlation distribution (*i.e.* *ρ* > 0.7 in normal samples). (B) The normal MMI-network (1738 nodes and 32375 edges) built in [86] starting from the expression data of normal breast tissues. Nodes in this network represent both mRNAs and lncRNAs; edges represent miRNAs that are mediating their interactions. Each pair of linked nodes fulfils two requirements: i. sensitivity correlation >0.3 and ii. one or more shared MREs, for each miRNA linking them. Colours correspond to different miRNAs.

The pipeline of the algorithm for searching putative ceRNAs and for building the MMI-network (Fig 1) in breast invasive carcinoma was presented in our previous work [86] and encompassed the following four steps: i. data collection and processing; ii. statistical analysis; iii. seed match analysis; iv. network building.

1. **Data collection and processing**
  Collections of tumour and normal expression data from high-throughput RNA- and miRNA-sequencing of breast invasive carcinoma were downloaded from the TCGA data portal [87, 88]. High-throughput sequencing data for both RNAs and miRNAs correspond to level 3 data (*i.e.* normalized expression data) given in terms of FPKM (*i.e.* fragments per kilobase of exon per million fragments mapped). The analysis was restricted to 72 individuals for which the complete sets of tumour and matched-normal profiles—for both short and long RNA-seq data—were available. Entries with more than the 10% of missing values were filtered out; coding versus non-coding RNAs based on Entrez gene identifiers and human annotation obtained from NCBI [120] were separated. The analysis was limited to those mRNAs with an available 3’ untranslated region (3’UTR) sequence at least equal to 500 nt in the curated UTRdb database [121]. All together, a total of 10492 mRNAs, 311 miRNAs, and 833 lncRNAs were analysed in [86].
2. **Statistical analysis**
  The top-correlated mRNA/lncRNA pairs in normal and cancer data sets were selected by setting in both cases the correlation threshold to the 99^th^ percentile of the corresponding overall correlation distribution (Fig 1A). Then, two regression models were built up: i. the expression profile of the mRNA is the dependent variable *X* and the expression profile of the miRNA is the explanatory variable *Z*; ii. the expression profile of the lncRNA is the dependent variable *Y* and the expression profile of the miRNA is the explanatory variable *Z*. The *X* and *Y* variables correspond to the top-correlated mRNA/lncRNA pairs.
  To infer the role of *Z* in mediating *X* − *Y* correlation, the partial correlation was computed:
  $$\begin{array}{c} \rho_{XY|Z}=\frac{\rho_{XY}-\rho_{XZ}\rho_{ZY}}{\sqrt{1-\rho_{XZ}^{2}}\sqrt{1-\rho_{ZY}^{2}}} \end{array}$$
  where *ρ*_*X*, *Y*_ is the Pearson correlation. Then, the *sensitivity correlation* S was defined as:
  $$\begin{array}{c} S=\rho_{XY}-\rho_{XY|Z} \end{array}$$ (1)
  The *XYZ* triplets with *S* > 0.3, corresponding to a drop of about the 30% in the correlation between XY when Z is removed, were selected. Finally, these triplets were restricted to those enriched in binding sites of the shared miRNA (hypergeometric test p-value <0.01).
3. **Seed match analysis**
  The minimal pairing requirement to predict a miRNA target recognition is a perfect match to positions 2 to 7 (6-mer miRNA seed) at the 5’-end of the mature miRNA sequence [122]. The miRNA seed sequences were obtained by mapping TCGA miRNA identifiers to miRBase [123]. Complementary DNA (cDNA) sequences (*i.e.* without introns) for lncRNAs were obtained querying the Ensembl [124] data portal through its R/Bioconductor [125] interface provided by the package *biomaRt* and by using Entrez gene identifiers [126]. For each 3’UTR sequence included in the dataset analysed in [86], all the occurrences matching the reverse-complement of the 6-mer seed for the miRNAs analysed were recorded. Similarly, for each lncRNA included the dataset analysed in [86] all the occurrences of short sites matching the reverse-complement of a miRNA seed in the entire transcript sequence were stored. The lists of coding and non-coding RNA identifiers used to retrieve corresponding sequences were built based on gene annotations obtained from the NCBI [120].
4. **Network building**
  The MMI-network both in normal and cancer tissues was built by integrating the results of statistical analysis and seed match analysis. Nodes in the networks represent mRNAs and lncRNAs with highly correlated expression profiles while edges represent miRNAs mediating their interactions. Concretely, linked nodes are required to meet three conditions: i. matching high values of the Pearson correlation between their expression profiles (*ρ* > 0.7); ii. matching high values of the sensitivity correlation (*S* > 0.3); iii. sharing binding sites for miRNAs (6-mer miRNA seed match).

### Raw data retrieval and processing

#### Data collection

Mapped read data (bam files) for the 72 patients (for which the complete sets of tumour and matched-normal profiles—for both short and long RNA-seq data—were available) analysed in [86] were downloaded from the TCGA [87] via controlled access (*i.e.* by using the TCGA dedicated software “gtdownload” to query via controlled access the restricted-access data repository). For each patient the relative two bam files corresponding to the breast tumour and normal sample are used as input for the Cufflinks software [127] in order to assemble transcripts and to estimate the relative abundances of these transcripts. As output formats the Cufflinks suite used FPKM tracking format. Then, we used Cuffmerge (a software included in Cufflinks) in order to merge together the 72 Cufflinks assemblies.

The PVT1 locus assembled by Cufflinks was compared with genome annotations for the same locus provided by Ensembl (release Homo sapiens GRCh37) by running the Cuffcompare utility and by careful inspection of the above assemblies and annotations on the UCSC genome browser.

Targeted reassembly of the PVT1 locus were performed using the Trinity software [128] with default parameters and digital normalization of the reads. To highlight possible differences between the healthy and tumour samples three independent assemblies were carried out, by using: a) all the reads mapping to the PVT1 locus from both cancer and normal tissue, b) only cancer reads, c) only reads from normal tissues. A UCSC genome browser track showing the main results of these analyses is available through this link: https://genome.ucsc.edu/cgi-bin/hgTracks?hgS_doOtherUser=submit&hgS_otherUserName=pantaleoM&hgS_otherUserSessionName=hg19_pone_S16_46501

While the overall agreement between the Trinity and Cufflinks assemblies is good, and both methods concur in recovering all of the PVT1 Refseq exons, we notice that some of exons predicted by Cufflinks are not supported by any of Trinity assemblies, and likely constitute false positives. Importantly the most prevalent isoforms reconstructed by Trinity are highly similar if not completely identical to the two most expressed PVT1 isoforms TCONS_147426 and TCONS_147501 predicted by Cufflinks, suggesting that these discrepancies in the assembly are not likely to play a major effect on isoforms abundance estimation. A differential alternative exon usage analysis (S1 Fig), performed by comparing the normalized reads counts distributions on the Refseq PVT1 exons, shows a striking pattern supporting the up-regulation of all the exons downstream of exon 5 in the tumour samples. This observation is highly consistent with our hypothesis that the up-regulation of PVT1 in tumour samples is mostly due to the up-regulation of isoforms of the gene devoid of the key exons exerting the sponge activity on miR-200 family members.

#### Statistical analysis

The PVT1 locus—assembled by the reference-based RNA-Seq transcriptome assembler Cufflinks using TCGA data of breast invasive carcinoma—is composed of 91 different isoforms (S2 Fig and S1 File). The FPKM normal and cancer data of these isoforms (S1 Table) were subjected to a pre-processing and filtering operation in order to reduce data noise and to select only the ones that show a statistically significant fold-changes between cancer and normal tissues (p-values of the Student’s t-test <0.05). Thus, the number of the PVT1 isoforms to analyse was trimmed to 17 isoforms.

#### Data classification

In order to classify the PVT1 isoforms on the basis of the FPKM data, we used the Principal Component Analysis (PCA) [129, 130]. PCA operates on a *n*-by-*p* data matrix *X*, whose rows correspond to the *n* observations and columns to the *p* variables. The representation of *X* in the principal component (PC) space is known as matrix of the principal component scores (S2 Table), whose rows correspond to observations and columns to the principal components (PCs). The transformation matrix from the old to the new coordinate system is known as matrix of factors (S2 Table), whose rows correspond to variables and columns to components. In this study, the original variables are the 72 patients and the observations are the PVT1 isoforms’ variations (*i.e.* the difference of the expression levels of the PVT1 isoforms between cancer and normal tissues).

## Results and discussion

Inspiring by our previous study presented in [86], in this manuscript we have investigated the specific conditions required for a ceRNA interaction network to occur. In particular, we thoroughly studied the intriguing phenomenon of the breakdown of the PVT1 functioning as sponge of the miR-200 family members in the breast invasive carcinoma by analysing the expression data of its multiple isoforms (S1 Table). The starting point of the present analysis, which complements the results obtained in [86], is represented by the investigation of the sensitivity correlation behaviour (Fig 1A), formerly inspected in [86] and whose mathematical expression is reported in Eq 1. This enables measuring the contribution of miRNAs in mediating the ceRNAs cross-talk and provides compelling clues on the nature of the ceRNA interactions, *i.e.* indirect (direct) interaction meaning that the ceRNAs communication is (is not) arbitrated by one or more microRNAs. As already mentioned in [86], in physiological conditions the value of the sensitivity correlation is almost zero, *i.e.* the Pearson correlation is equal to the partial correlation, leading to the expected conclusion that, in normal breast tissues, the majority of the miRNAs is not arbitrating the cross-talk between long non-coding RNAs and coding RNAs. Thus, the observed high correlations between the expression profiles of the top-correlated lncRNA/mRNA pairs could be presumably ascribable to a common transcriptional regulatory mechanism, rather than to a post-transcriptional regulation program orchestrated by shared miRNAs. Nevertheless, a small pool of miRNAs appears as responsible of the vertical light stripes that unexpectedly stand out from the prevailing red colour of the background of Fig 1A. Hence, these miRNAs can be reasonably envisaged as the mediators of the interactions between all the highly correlated pairs in the normal breast samples. Among them, there are all members of the miR-200 family, whose importance in breast cancer is well-known and is related to the epithelial-mesenchymal transition. This pattern completely disappears in cancer [86] to give way to the activation of a different ceRNA landscape. This “on/off” switch from normal to cancer, and *vice-versa*, leads to the inference of a marked rewiring in the ceRNA program between normal and pathological breast tissue that confers an interesting character to ceRNAs as potential oncosuppressive, or oncogenic, protagonists in cancer.

**Fig 2:**
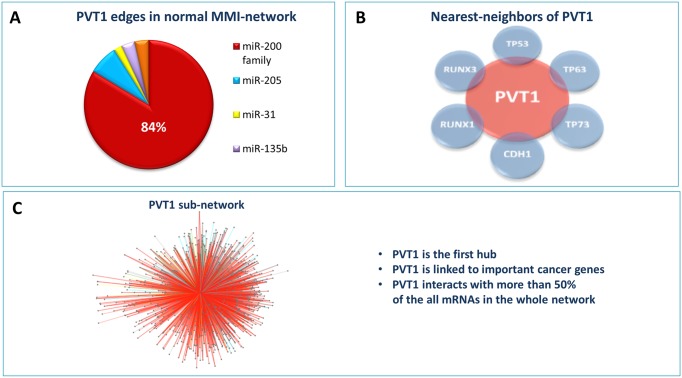
PVT1: the first hub of the normal MMI-network. (A) The percentage of the miRNAs sponged by PVT1 with respect to all of its links. More than the 80% corresponds to the miR-200 family members. (B) Some nearest neighbours of PVT1 that are well-known cancer genes as the members of the p53 family or the members of the RUNX family, as well as E-cadherin. (C) The sponge interactions sub-network of PVT1. It consists of 2169 edges and 753 nodes, more than the 50% of all the mRNAs in the whole normal MMI-network.

Using the sensitivity correlation and the results of the seed match analysis, summarised in Materials and Methods section, the MMI-network was built in both the physiological and pathological condition of human breast cancer dataset analysed in [86]. Nodes of these network are lncRNAs and mRNAs that are competing for miRNA binding and links are the “bone of contention” miRNAs (Fig 1B). The lncRNA PVT1 with its 2169 edges represents the first hub (*i.e.* the node with the largest number of links or the highest degree in the network) in the normal-MMI-network. It is connected to 753 different mRNAs (∼ 50% of total mRNAs in the network) and the miR-200 family members are arbitrating over the 80% of these interactions (Fig 2A). Moreover, PVT1 has as nearest neighbours some of the well-known cancer genes (Fig 2B) and is connected to 753 different mRNAs representing more than the 50% of all the mRNAs in the whole normal MMI-network (Fig 2C).

**Fig 3:**
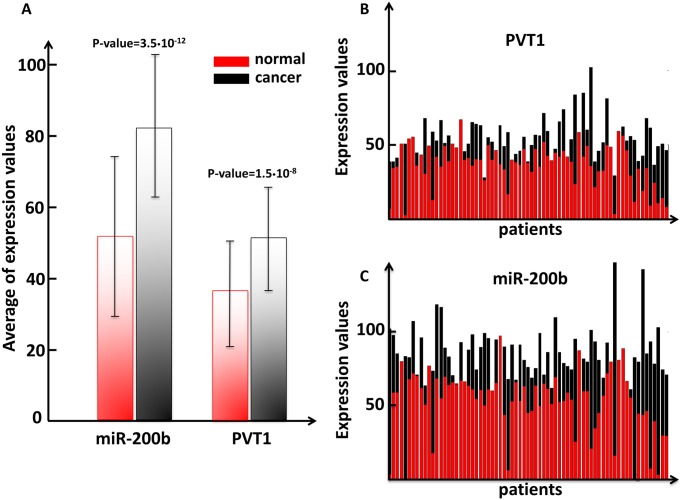
PVT1 and miR-200b expression levels in human breast cancer tissues. The main actors of the normal MMI-network are the miR-200 family members and the long non-coding PVT1. Both of them appear to be up-regulated in breast cancer tissues with respect to normal breast tissues. (A) The mean and the standard deviation for the long non-coding PVT1 and for the miR-200b, one member of the miR-200 family, in normal (red boxes) and cancer samples (black boxes). In figure the p-values resulting from the statistical hypothesis Student’s t-test are reported. (B-C) Level 3 (*i.e.* normalized expression data) IlluminaHiSeq expression data of PVT1 and the miR-200b for all patients given in terms of FPKM (*i.e.* fragments per kilobase of exon per million fragments mapped). Red boxes correspond to normal tissues while black boxes correspond to cancer tissues.

By analysing the expression profiles of PVT1, obtained from the dataset studied in [86], over all the patients, we found that it is up-regulated in breast cancer tissues both as mean value (Fig 3A) and individually on each patient, regardless of the breast cancer subtypes (Fig 3B). This up-regulation is counteracted by a similarly, but even more significant, overexpression of the miR-200 family members (see Fig 3A and 3C for the representative case of the miR-200b). The question, then, arises: if PVT1 and the miR-200 family are both up-regulated in cancer, why PVT1 stops working as sponge in cancer?

**Fig 4:**
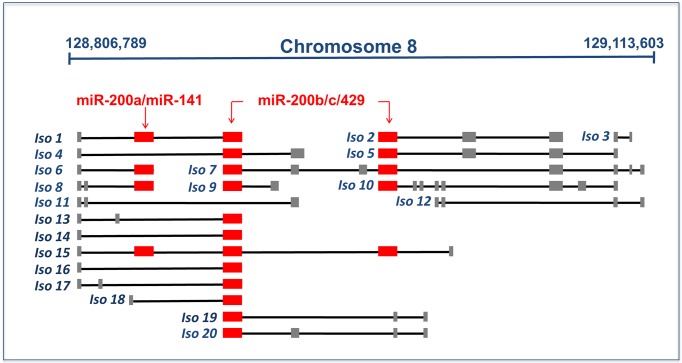
Sketch of the PVT1 locus in humans. Model of PVT1 genomic locus as reconstructed by Cufflinks (S1 File) spans across a genome interval of over 300 kb (*i.e.* bases 128,806,789-129,113,603 within the February 2009 human genome build GRCh37/hg19) on the forward strand of chromosome 8. The large PVT1 locus gives rise to 91 different variants (S2 Fig) according to raw RNA-seq data of TCGA for breast invasive carcinoma. The isoform names correspond to an increasing symbolic numbering and not to the actual nomenclature of the PVT1 variants. Lines represent introns and boxes (red and grey) represent exons. Red boxes correspond to the binding sites for the miR-200 family members. Note that some isoforms lack such binding sites (*e.g.* Iso11 and Iso12).

The analysis of the PVT1 genomic locus showed the existence of multiple isoforms (Fig 4 and S2 Fig) representing all the possible configurations: hosting the binding site for some (*e.g.* Iso6 or Iso7 in Fig 4) or all members of the miR-200 family (*e.g.* Iso1 in Fig 4); missing the binding site (*e.g.* Iso11 and Iso12 in Fig 4). This consideration together with the observed synchronised up-regulation of the PVT1 gene and the miR-200 family members encouraged us to hypothesize different scenarios that could be in principle compatible with the ceasing of the PVT1 sponge activity in breast cancer tissues. From one hand, the absence in two PVT1 isoforms of the exon where the MREs for the all members of the miR-200 family reside could lead to support the hypothesis of a preferential expression in cancer tissues of these two isoforms, thus justifying the lack of the miRNA/target interaction with a consequent breakdown of the PVT1 ceRNA activity (*i.e.* the exon skipping mechanism). From the other hand, the observation of a simultaneous up-regulation of the PVT1 gene and the miR-200 family members could lead to support the alternative hypothesis of different relative concentrations between each isoform and the miR-200 family members. According to that, a substantial decrease in cancer tissues of the relative variation of the isoform harbouring the binding site for one or more members of the miR-200 family could be due to a huge increase of the miR-200 family associated with a moderate growth in cancer of the expression levels of this PVT1 isoform. This situation, completely different from what occurs in normal tissues where the miRNA/target concentrations are comparable, could give reason of the PVT1 cease-activity as ceRNA in cancer (*i.e.* a titration mechanism).

**Fig 5:**
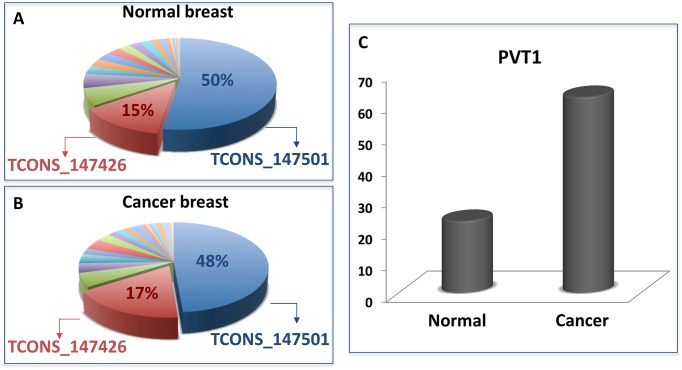
PVT1 isoforms in normal and cancer breast tissues. The PVT1 abundance in terms of its isoforms in normal (A) and cancer tissues (B) of breast invasive carcinoma. The percentage are calculated with respect to the total abundance of PVT1 in normal and cancer tissues (S3 Table). In both panels, the red slice corresponds to the isoform (TCONS_147426) with seed match for the miR-200b/200c/429 cluster and the blue slice corresponds to the isoform (TCONS_147501) lacking the binding site for any member of the miR-200 family. (C) The average of PVT1 abundance on all the isoforms both in normal and cancer tissues.

To shed light on which of the two hypothesised mechanisms lies the origin of the PVT1 stoppage as sponge, we looked at the PVT1 abundance in terms of its isoforms and we found that in both normal (Fig 5A and S3 Table) and cancer tissues (Fig 5B and S3 Table) only two isoforms represent the biggest slices: the first largest slice—which corresponds to the 50% (48%) of the PVT1 total abundance in normal (cancer) breast samples—represents the isoform missing the binding site for the miR-200 family (TCONS_147501); the second largest slice—which corresponds to the 15% (17%) of the PVT1 total abundance in normal (cancer) breast samples—represents the isoform hosting the binding site for the miR-200b/200c/429 cluster (TCONS_147426). Overall, both in normal and cancer tissues the two isoforms TCONS_147501 and TCONS_147426 represent about the 65% of the total abundance of PVT1 (S3 Table). Moreover, PVT1 resulted up-regulated also in terms of its total isoforms abundance (Fig 5C), confirming the result obtained at gene level (Fig 3A).

The observation that both the isoforms, with and without the exons where the MREs of the miR-200 family memebrs reside, resulted expressed in both cancer and normal breast tissues undermine the truthfulness of the hypothesis rested on the exon skipping mechanism and corroborates the proposal based on the relative concentrations of the PVT1 isoforms and the miR-200 family members.

Thus, in order to sift through the validity of a titration mechanism, we performed the principal component analysis using the feature abundance levels of all the PVT1 isoforms across samples (S1 Table). The aim of PCA is to determine the principle axes of the abundance variation and to separate the isoforms according to this feature. This is achieved through a reduction of the space dimensionality that transforms a high-dimensional dataset—where the dimension of the space is equal to the linear independent variables (*i.e.* patients)—into a smaller-dimensional subspace—where the dimension of the space is equal to number of PCs that are able to explain the first 100% of the cumulative distribution of the explained variance of the data. The first step of this analysis is to draw a new axis representing the direction of maximum variation through the data (the first PC). Next, another axis is added orthogonal to the first and positioned to represent the next highest variation through the data (the second PC), and so on.

**Fig 6:**
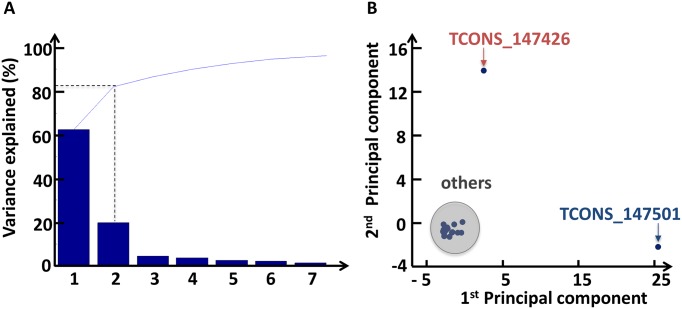
Principal component analysis. (A) The percent variability explained by each principal component (S2 Table). It is a type of chart, called Pareto chart, that contains both bars and a line graph, where individual values are represented in descending order by bars, and the line represents the cumulative total value. In particular, the y-axis represents the percentage of the data variance explained by each principal component, whereas the x-axis represents the principal components that are able to explain the first 100% of the cumulative distribution. The PCA is performed using the variations of all the isoforms between normal and cancer tissues. Two components explain more than the 80% of the variance of the data. (B) The scatter plot (score plot) of the projection of the original data (*i.e.* the variations of all the isoforms between normal and cancer tissues) onto the first two PCs; the x-axis contains the first PC while the y-axis contains the second PC (S2 Table). In this plot, it is possible to group isoforms in three classes: the isoform missing the binding site for the miR-200 family members (blue isoform, TCONS_147501), the isoform with the seed match for the miR-200b/200c/429 cluster (red isoform, TCONS_147426), and all the others. The first PC, which explains about the 60% of the variance in the original data, is able to separate the variation of the blue isoform from the others; the second PC, which explains about the 20% of the variance in the original data, is able to separate the variation of the red isoform from the others.

We found that two PCs are able to explain more than the 80% of the variance of the data (Fig 6A and S2 Table). In order to understand the meaning of these two PCs, we drew the score plot (Fig 6B and S2 Table) and found that the first PC is able to separate the contribution of the isoform missing the binding site for any members of the miR-200 family from the others, while the second PC is able to separate the contribution of the isoform hosting the binding site for the miR-200b/200c/429 cluster from the others.

This suggests the following argument of plausibility of the PCA analysis results: the first PC, which explain by alone about the 60% of the total variance of the analysed data (S2 Table), corresponds to the variation of the isoform that, missing the binding site, does not interact with the miR-200 family; while the second PC, explaining by alone about the 20% of the total variance of the analysed data (S2 Table), represents the variation of the isoform that, hosting the binding site for the miR-200b/200c/429 cluster, could be act as competitors of the targets of these miRNAs. Overall the variation between cancer and normal tissues of these two isoforms accounts for more than the 80% of the variance of the data (Fig 6A and S2 Table).

**Fig 7:**
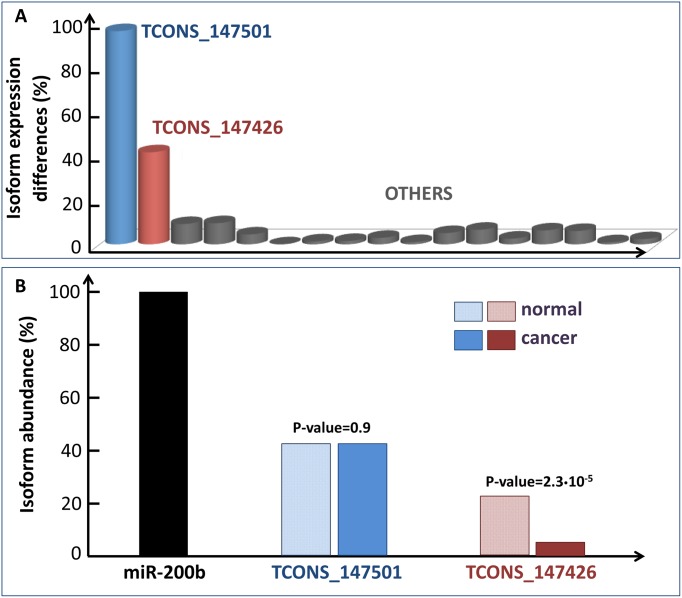
PVT1 isoforms variation. (A) The variations between cancer and normal tissues of all the PVT1 isoforms with respect to the variation of the blue isoform lacking the binding site for the miR-200 family members (S4 Table). The red and blue isoforms are the only isoforms that change. (B) The ratio between the abundance of the blue and red isoform with respect to the miR-200b in both normal (striped rectangle) and cancer tissues (full boxes). The p-values resulting from the statistical hypothesis Student’s t-test are reported. The ratio between the blue isoform and the miR-200b does not change, while the ratio between the red isoform and the miR-200b shows a drastic fall in cancer tissues.

Studying the variation of each PVT1 isoform between normal and cancer breast tissues with respect to the variation of TCONS_147501, the results of PCA seems to be confirmed (Fig 7 and S4 Table): the isoform harbouring the binding site for the miR-200b/200c/429 cluster and the isoform missing the binding site for any member of the miR-200 family, are the only isoforms that change (Fig 7A).

**Fig 8:**
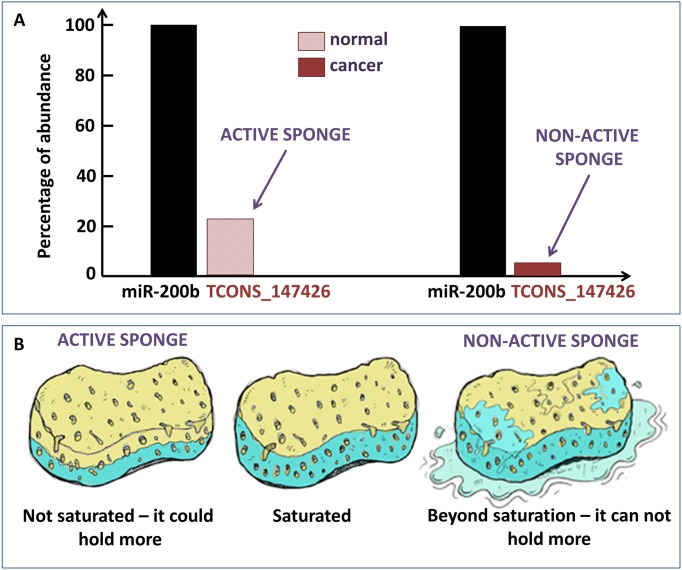
Sponge mechanism. (A) The ratio between the abundance of the red isoform (harbouring the binding site for the miR-200b/200c/429 cluster) over the abundance of the miR-200b in both normal (striped rectangle) and cancer tissues (full boxes). (B) Our hypothesis for the breakdown of the sponge mechanism in breast cancer orchestrated by PVT1 and the miR-200 family members. We speculate that in the normal tissues only the red isoform of PVT1 gene acts as sponge regulator of the miR-200 family members. In cancer tissues it stops working as sponge since its concentration is much lower than the concentration of the miR-200 family members (here is reported only the case of miR-200b). It is like in the case of a real sponge: before saturation the sponge can hold more water, beyond saturation—there is too much water—the sponge can’t hold more.

Thus, we considered only these two isoforms (S2 File) and evaluated the ratio between the abundance of each one with respect to one representative member of the miR-200b/200c/429 cluster (*i.e.* miR-200b) in both normal and cancer tissues. For the TCONS_147501 isoform (missing the binding site) this ratio does not change between normal and breast cancer tissues, while in the case of the TCONS_147426 isoform (harbouring the binding site) this ratio shows a drastic decrease from normal to cancer tissues (Fig 7B). We speculate that the TCONS_147426 isoform acts as sponge regulator of the miR-200b in normal breast tissues, while the sponge mechanism is broken down in cancer tissues because this isoform shows a much lower concentration with respect to the miR-200b (Fig 8A). Informally speaking, such a sponge mechanism works as a real sponge: before saturation the sponge can hold more water, beyond saturation—there is too much water—the sponge can not hold more (Fig 8B).

So, our analysis supports the hypothesis that the “on/off” switch from normal to cancer state of the PVT1 sponge activity is mostly due to the variation of the relative concentration of PVT1 isoform hosting the binding site for the miR-200b/200c/429 cluster.

## Conclusion

Starting from the results presented in [86]—where we analysed the complex interactions among mRNAs, long non-coding RNAs, and microRNAs in breast invasive carcinoma—here we investigated the mechanism underlying the marked rewiring of the sponge program between normal and cancer tissues. In particular, the analysis of the normal miRNA-mediated interactions network, built in [86], pointed out how the main actors of this rewiring were PVT1 and the miR-200 family members. Specifically, PVT1 emerged as a putative ceRNA modulating the activity of all members of the miR-200 family on their target mRNAs, which are well-known to be drastically involved in breast cancer morphogenesis and development. Interestingly, such a sponge mechanism resulted completely abolished in cancer tissues, although both PVT1 and the miR-200 family members appeared up-regulated in the pathological condition. Thus, processing the raw data from TCGA, which provided the abundance of the multiple isoforms generated by the PVT1 genomic locus, we tried to grasp the rational behind the turning off of this sponge mechanism. In particular, the principal component analysis suggested that the variations between cancer and normal breast tissues of all PVT1 isoforms can be explained by only two principal components: one corresponding to the isoform harbouring the binding site for the miR-200b/200c/429 cluster and the other one representing the isoform missing the binding site for any member of the miR-200 family members. Moreover, comparing the relative expression levels of these two isoforms both in normal and cancer tissues with respect to the ones of the one representative member of the miR-200b/200c/429 cluster (*i.e.* miR-200b), we found a drastic drop, in the pathological condition, in the relative concentration of the PVT1 isoform hosting the binding site for the miR-200b. The drastic change observed in the sponge program, which is suggestive of a marked ceRNA rewiring that characterizes the cancer state, could support the testable hypothesis of a titration mechanism regarding the two main isoforms of PVT1 and the miR-200 family members.

## Supporting information

S1 Fig Differential alternative PVT1 usage analysis. This figure shows the results of a differential alternative exon usage analysis, performed by comparing the normalized reads counts distributions on the Refseq PVT1 exons. It shows a striking pattern supporting the up-regulation of all the exons downstream of exon 5 in the tumour samples. This observation is highly consistent with our hypothesis that the up-regulation of PVT1 in tumour samples is mostly due to the up-regulation of isoforms of the gene devoid of the key exons exerting the sponge activity on miR-200 family members.
(PNG)

S2 Fig Visualisation of PVT1 genomic locus in human. This figure shows the 91 PVT1 isoforms (*i.e.* bases 128,806,789-129,113,603 within the February 2009 human genome build GRCh37/hg19) visualised within the UCSC Genome browser (https://genome.ucsc.edu/) and assembled by the reference-based RNA-Seq transcriptome assembler Cufflinks by using the TCGA breast invasive carcinoma dataset.
(PNG)

S1 File Reconstruction of PVT1 genomic locus in human. This file contains PVT1 gene annotations in GTF (Gene Transfer Format) format provided by the reference-based RNA-Seq transcriptome assembler Cufflinks. This file is a simple tab-delimited text file for describing genomic features and it can be uploaded to a genome browser such as the UCSC Genome browser (https://genome.ucsc.edu/) in order to obtain the S2 Fig.
(GTF)

S2 File Sequences of two PVT1 isoforms. This file contains the full genome sequences (in FASTA format) of the two PVT1 isoforms that mostly change between normal and cancer tissues: TCONS_147501 (missing the binding site for miR-200 family members) and TCONS_147426 (harbouring the binding site for the miR-200b/200c/429 cluster).
(FA) Click here for additional data file.

S1 Table PVT1 isoforms expression levels. This table reports the FPKM values of PVT1 isoforms across normal and cancer breast tissues in separate and accordingly named sheets.
(XLSX)

S2 Table Principal Component Analysis. This table reports the results of the principal component analysis, in separate and accordingly named sheets: first sheet) the eigenvalues of the covariance matrix of the *n*-by-*p* data matrix *X*, whose rows correspond to observations (*i.e.* isoforms’ variations that are the difference of the expression levels of the PVT1 isoforms between cancer and normal tissues) and columns to variables (*i.e.* patients), the variance accounted for by each component, and the cumulative function; second sheet) the matrix of the principal component scores, whose rows correspond to observations and columns to components; third sheet) the matrix of factors, whose rows correspond to variables and columns to components.
(XLSX)

S3 Table PVT1 isoform abundance. This table reports the percentage of PVT1 abundance, showed in Fig 5, in terms of its isoforms both in normal and cancer tissues of TCGA breast invasive carcinoma.
(XLSX)

S4 Table PVT1 isoforms variation. This table reports the variations between cancer and normal tissues of the expression levels of all the PVT1 isoforms with respect to the variation of the TCONS_147501 isoform lacking the binding site for the miR-200 family members, showed in Fig 7A.
(XLSX)
