# Supplementary figures and images for: Role of the long non-coding RNA PVT1 in the dysregulation of the ceRNA-ceRNA network in human breast cancer
Source: PLoS One. 2017 Feb 10;12(2):e0171661. doi: 10.1371/journal.pone.0171661 (PMC5302781; doi:10.1371/journal.pone.0171661)

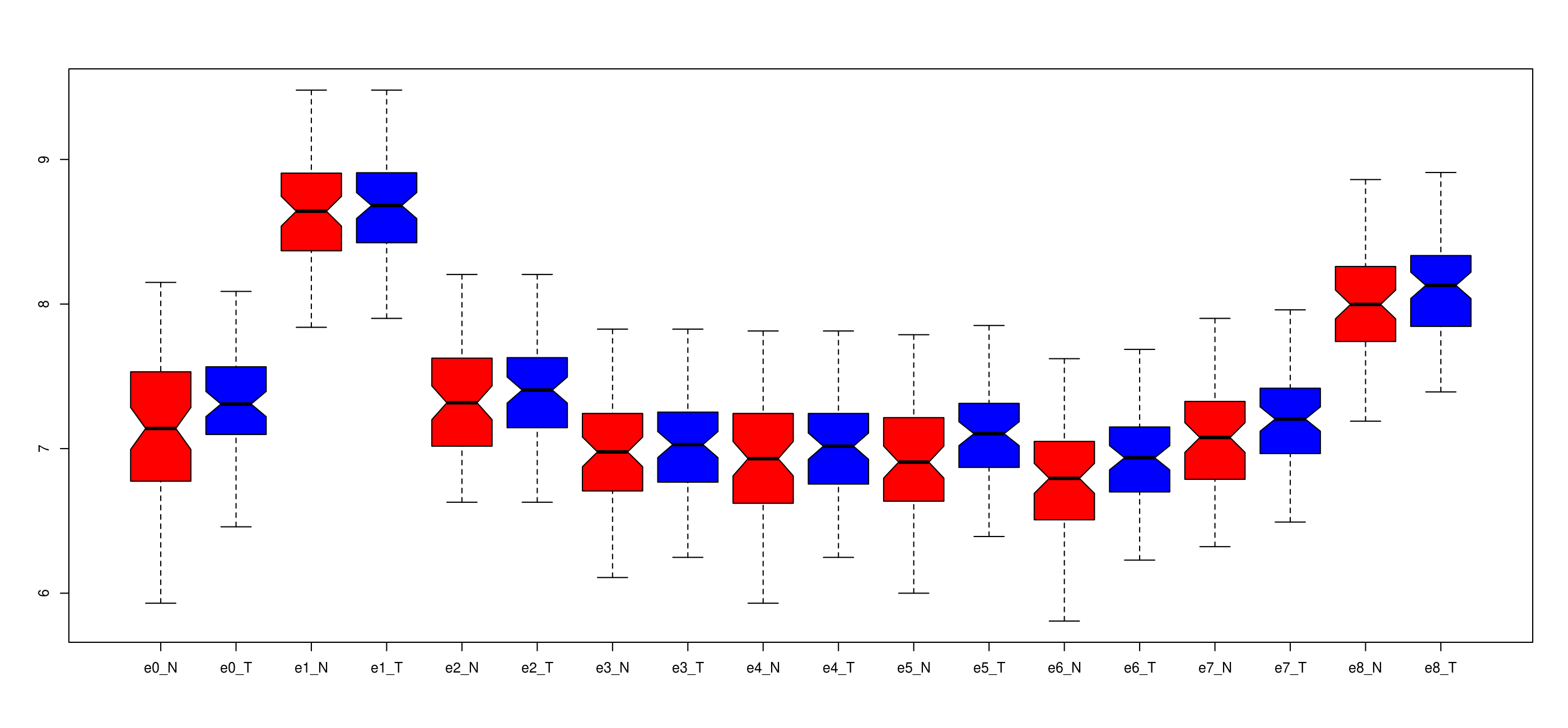

Supplement: S1 Fig — This figure shows the results of a differential alternative exon usage analysis, performed by comparing the normalized reads counts distributions on the Refseq PVT1 exons. It shows a striking pattern supporting the up-regulation of all the exons downstream of exon 5 in the tumour samples. This observation is highly consistent with our hypothesis that the up-regulation of PVT1 in tumour samples is mostly due to the up-regulation of isoforms of the gene devoid of the key exons exerting the sponge activity on miR-200 family members. (PNG) [file pone.0171661.s001.png]

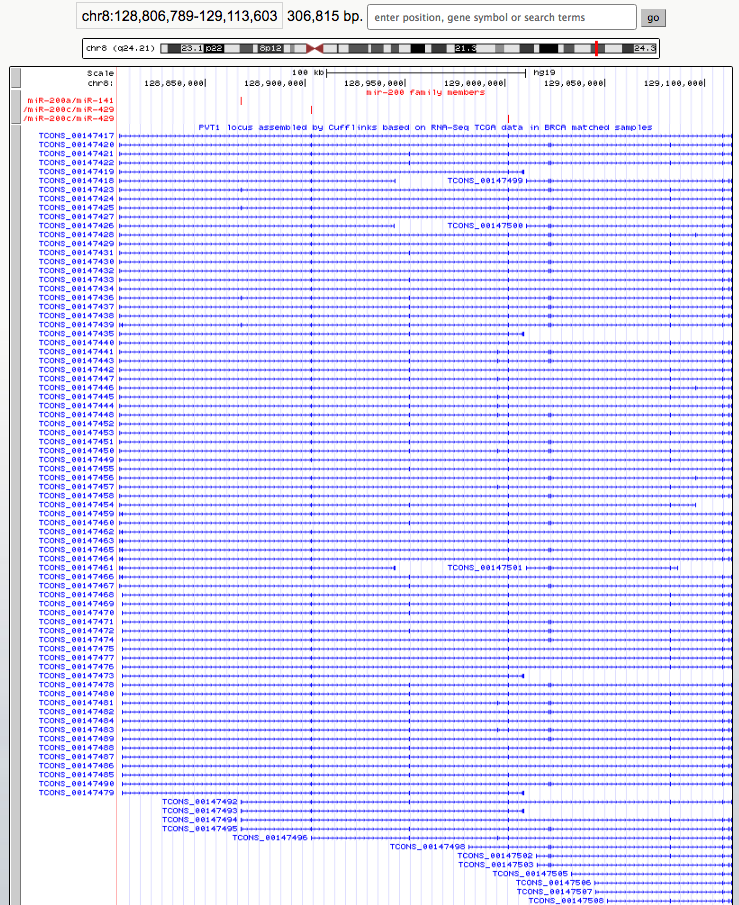

Supplement: S2 Fig — This figure shows the 91 PVT1 isoforms (i.e. bases 128,806,789-129,113,603 within the February 2009 human genome build GRCh37/hg19) visualised within the UCSC Genome browser (https://genome.ucsc.edu/) and assembled by the reference-based RNA-Seq transcriptome assembler Cufflinks by using the TCGA breast invasive carcinoma dataset. (PNG) [file pone.0171661.s002.png]
